# Opioid Crisis—An Emphasis on Fentanyl Analogs

**DOI:** 10.3390/brainsci10080485

**Published:** 2020-07-27

**Authors:** Kabirullah Lutfy

**Affiliations:** College of Pharmacy, Western University of Health Sciences, Pomona, CA 91766, USA; klutfy@westernu.edu

**Keywords:** opioids, novel fentanyl derivatives, analgesia, overdose, addiction, respiratory arrest, opioid receptors, opioid crisis

## Abstract

Opioids are the mainstay for the management of moderate to severe pain. However, their acute use is associated with several side effects, ranging from nausea, itching, sedation, hypotension to respiratory depression, and death. Also, chronic use of these drugs can lead to the development of tolerance, dependence, and eventually addiction. The most serious side effect, lethality due to opioid-induced overdose, has reached the level of national emergency, i.e., the opioid crisis, which is now the forefront of medicine. In a detailed review (Novel Synthetic Opioids: The Pathologist’s Point of View), Frisoni and colleagues have discussed the side effects of novel licit and illicit fentanyl derivatives, as well as the related compounds which are more potent and faster acting than morphine and other conventional opioids (Frisoni, et al., 2018). These drugs affect the central nervous system (CNS) and can promote the development of addiction due to the quick rush they induce because of their faster entry into the brain. These drugs also arrest the cardiovascular and pulmonary systems, increasing the chance of respiratory arrest, leading to opioid-induced overdose morbidity and mortality. The respiratory arrest induced by opioids can be potentiated by other CNS depressants, such as alcohol or benzodiazepines, and therefore may occur more frequently in polydrug users. Therefore, the use of these newer fentanyl derivatives as well as other fast acting opioids should be avoided or limited to specific cases and must be kept out of the reach of children and adolescents who are more vulnerable to become addicted or overdose themselves.

Opioids are used for the treatment of moderate to severe pain. Opioids exert their actions via the opioid receptors, namely the mu, delta, and kappa opioid receptors. These receptors belong to the G-protein-coupled receptors (GPCRs) family which consists of seven transmembrane domains and an amino (N)-terminal located outside the cell membrane and a carboxyl (C)-terminal inside the cell. These receptors mediate the actions of endogenous opioids, such as beta-endorphin, enkephalins, and dynorphins, as well as exogenous opioids such as morphine, fentanyl, codeine, heroin, etc. Opioid receptor antagonists also bind to these receptors and block the actions of opioid agonists. At the cellular level, opioids exert inhibitory effects because opioid receptors are coupled to Gi/o proteins, leading to the inhibition of adenylyl cyclase and calcium channels, as well as the activation of the potassium channels. These cellular events lead to the hyperpolarization of cells as well as to the reduction of neurotransmitter release from the presynaptic terminals of cells containing opioid receptors or making any type of synaptic connection with the opioid-containing cells. Although opioids reduce neurotransmitter release, there are occasions where opioids could increase neurotransmitter release, e.g., the opioid-mediated release of dopamine in the nucleus accumbens, but this effect occurs due to disinhibition of dopaminergic neurons in the ventral tegmental area [1], which is thought to play an important role in the rewarding action of opioids, as well as in the initiation and maintenance of addiction (for reviews, see [2,3,4,5,6]). Among other properties, it is believed that any drug that enters the brain faster and increasing the level of dopamine rapidly, having a short duration of action, may be more addicting than a slower acting drug. In this regard, it is important to list heroin, fentanyl, and its newer derivatives, which may be the cause of the opioid crisis, especially when these drugs are combined with other CNS depressants [7].

Opioid analgesics exert inhibitory effects on nociceptive processing, leading to a potent analgesic effect. However, the chronic use of these drugs leads to tolerance and dependence. These drugs also reduce gastrointestinal motility, as well as the cardiovascular and respiratory systems, leading to constipation and respiratory arrest, respectively, the latter being the main culprit of the opioid crisis, which is currently at the forefront of medicine [8,9]. Therefore, intense research has been underway to develop novel opioids that could reduce pain with limited side effects. However, earlier attempts led to the discovery of heroin, fentanyl, and more recently to the generation of newer fentanyl derivatives, which are useful as analgesics and preanesthetic agents, but they also possess dangerous side effects due to their faster onset of action and greater potency than morphine. The use of prescription opioids has fueled the opioid epidemic to the level of a national emergency. In 2017 alone, there were more than 70,000 deaths due to opioid overdose. Although this number is lower in Canada, it reached 4000 deaths due to opioid overdose but in some parts of Canada, it is as bad as the US [9]. However, the take-home kit containing naloxone saved many lives [9]. Nevertheless, the latter drug needs to be administered early enough to save lives.

Fentanyl and novel synthetic licit and illicit fentanyl derivatives have significantly contributed to these deaths due to opioid overdose because of their narrow therapeutic index [7]. Besides fentanyl and its derivatives, other opioids that were used as recreational drugs and caused morbidity and mortality were MT-45, AH-7921, and U-47700 [7]. The main CNS side effect reported was cerebral edema. The deleterious cardiovascular and pulmonary side effects were petechiae due to asphyxia, respiratory depression, chest wall rigidity, and apnea [7]. Thus, there is a critical need to develop novel medications that possess analgesic effects with limited cardiovascular and pulmonary side effects, as well as reduced rewarding actions and dependence liability compared to the currently used opioid analgesics.

Recently, among the other approaches, drugs that activate the opioid receptor-like receptor (ORL1, also known as NOP) another member of the opioid receptor family, with concomitant activity at the mu opioid receptors (MOP), known as bifunctional NOP/MOP agonists, have been shown to provide a potent analgesic profile [10]. Indeed, the therapeutic potential of this bifunctional approach is currently being validated in the clinic for the first time with cebranopadol, the first NOP/MOP bifunctional agonist small molecule [11,12] to enter clinical trials for the management of pain [13]. However, cebranopadol has equipotent efficacy at NOP (100% agonist) and MOP (98% agonist efficacy), and while it exerts potent antinociceptive effects in many rodent models of pain, it also shows a morphine-like discriminative stimulus in rats [14], suggesting that the drug has the potential for at least abuse, if not overdose. Thus, an optimum balance between NOP and MOP efficacy is desirable to generate effective and safer analgesics. Along this line, exciting data in nonhuman primates showed that a bifunctional NOP/MOP partial agonist AT-121 exerted analgesia which was superior to morphine, and did not induce respiratory depression or itch and displayed no tolerance or reinforcing effects. Until the availability of such compounds in clinical settings, physicians should be wary about the deleterious cardiovascular and respiratory side effects of currently used opioids, which are the main culprits of opioid-induced morbidity and mortality. Furthermore, more restrictions should be placed on the availability of licit and illicit fentanyl derivatives on the internet and street and must be kept out of the reach of children and adolescents to avoid diversions.
